# Coronavirus disease 2019 (COVID-19) in pediatric patients with autoimmune disorders

**DOI:** 10.1007/s00431-023-04958-6

**Published:** 2023-04-19

**Authors:** Parniyan Sadeghi, Parmida Sadat Pezeshki, Nima Rezaei

**Affiliations:** 1 School of Medicine, Shahid Beheshti University of Medical Sciences, Tehran, Iran; 2 Network of Immunity in Infection, Malignancy and Autoimmunity (NIIMA), Universal Scientific Education and Research Network (USERN), Tehran, Iran; 3 School of Medicine, Tehran University of Medical Sciences, Tehran, Iran; 4 Cancer Immunology Project (CIP), Universal Scientific Education and Research Network (USERN), Tehran, Iran; 5 Research Center for Immunodeficiencies, Children’s Medical Center Hospital, Tehran University of Medical Sciences, Dr. Qarib St, Keshavarz Blvd, 14194 Tehran, Iran; 6 Department of Immunology, School of Medicine, Tehran University of Medical Sciences, Tehran, Iran

**Keywords:** COVID-19, Autoimmune disease, Pediatrics, Immunomodulatory agents, Immunosuppression

## Abstract

Coronavirus disease 2019 (COVID-19) infection in pediatric patients with autoimmune disorders is an area of particular concern since autoimmune diseases can increase the risk of complications from the virus. However, as the infection rates were significantly higher in adults compared to children, this at-risk group of children was relatively underrepresented in COVID-19 research. The underlying inflammatory basis of autoimmune diseases and medications that affect the immune system, such as corticosteroids, could increase the risk of severe infection in this group of patients. COVID-19 could reportedly lead to a variety of alterations in the immune system. These alterations are plausibly dependent on the underlying immune-mediated diseases or prior use of immunomodulatory drugs. Patients administrating immunomodulatory agents, especially those with severe immune system dysregulation, can experience severe symptoms of COVID-19. Nonetheless, receiving immunosuppressive medications can benefit patients by preventing cytokine storm syndromes and lung tissue damage, threatening outcomes of COVID-19.

*Conclusion:* In this review, we sought to evaluate the currently available literature on the impact of autoimmune disease and its related therapeutic approaches on the COVID-19 infection course of disease in children and reflect on the gaps in the evidence and the need for further research in this field.

**Table:** 

**What is Known:** • The majority of children infected with COVID-19 demonstrate mild to moderate clinical manifestations compared to adults, whereas those children with pre-existing autoimmune conditions are at a greater risk for severe symptoms. •There is currently limited understanding of the pathophysiology and clinical outcomes of COVID-19 in pediatric patients with autoimmune disorders due to scattered reports and inadequate evidence.
**What is New:** • Generally, children with autoimmune disorders have more unfavorable outcomes than healthy children; yet, the severity is not extreme, and is highly dependent on their autoimmune disease type and severity, as well as the medication they are taking.

**What is Known:**

• The majority of children infected with COVID-19 demonstrate mild to moderate clinical manifestations compared to adults, whereas those children with pre-existing autoimmune conditions are at a greater risk for severe symptoms.

•There is currently limited understanding of the pathophysiology and clinical outcomes of COVID-19 in pediatric patients with autoimmune disorders due to scattered reports and inadequate evidence.

**What is New:**

• Generally, children with autoimmune disorders have more unfavorable outcomes than healthy children; yet, the severity is not extreme, and is highly dependent on their autoimmune disease type and severity, as well as the medication they are taking.

## Introduction

On January 7, 2020, a new coronavirus was discovered in lung fluid from a patient with pneumonia-like symptoms in Wuhan, China, using meta-transcriptomic sequencing [1]. Since then, the severe acute respiratory syndrome coronavirus 2 (SARS-CoV-2), the pathogen that causes coronavirus disease 2019 (COVID-19), has produced extreme levels of morbidity and mortality around the world [2]. In March 2020, the World Health Organization (WHO) proclaimed the novel coronavirus outbreak a pandemic [3]. SARS-CoV-2 is an enveloped, positive-sense, single-stranded ribonucleic acid (RNA) virus belonging to the Coronaviridae family [4]. It is the seventh coronavirus to infect humans, following the discovery of SARS and Middle East respiratory syndrome viruses (MERS) [5]. The virus attacks the lower respiratory tract and causes pneumonia in humans. The symptoms are milder than those of SARS or MERS, but the disease can eventually become lethal due to hyperinflammation and respiratory failure [6].

COVID-19 has been detected in 759,408,703 people globally, with 6,866,434 deaths reported by WHO on 7 March 2023 [7]. SARS-CoV-2 cases in children under 18 are estimated to account for 2%–5% of all SARS-CoV-2 cases worldwide [8]. Albeit, it must be considered that most children with COVID-19 infection are asymptomatic or have non-specific mild to moderate symptoms. Moreover, due to the prioritization of broad COVID-19 testing for adults and patients with severe symptoms, the reported incidence of SARS-CoV-2 infection in children might be underestimated [9]. However, by June 10, 2022, over 13,400 deaths in people under the age of 20 were reported, with 58% of deaths occurring among teenagers aged 10 to 19, and 42% among those aged 0 to 9 years old [10].

Fever was the most common symptom, followed by cough, nasal symptoms, diarrhea, and nausea/vomiting in COVID-19-infected children [11]. Comorbidities, including obesity, diabetes, heart diseases, chronic lung diseases, epilepsy, and immunocompromised conditions, were identified as risk factors for higher morbidity and mortality of COVID-19 among children [12, 13].

Patients with autoimmune diseases are also reported to be at a higher risk of COVID-19 infection [14]. This could be ascribed to the regular use of glucocorticoids and other anti-rheumatoid agents in this group of patients [15]. Research also found that genetic and epigenetic factors related to autoimmune diseases like lupus, rheumatoid arthritis, type 1 diabetes, and Graves' disease might increase the risk and severity of SARS-CoV-2 infections [16–18]. Investigations of COVID-19 infection in individuals with autoimmune diseases and the pathologic effect of these conditions on COVID-19 severity have yet to be small in scale and generally region-specific. As a result, the effects of COVID-19 on patients with autoimmune diseases are still unknown [19–21]. In addition, because of the low prevalence of pediatric cases, clear conclusions about the natural history of COVID-19 in pediatric patients with autoimmune diseases have been even more difficult to obtain [8].

Accordingly, the present study was designed to comprehensively explore what is currently known and what knowledge we still lack about the pathophysiology of COVID-19, diagnosis, prognosis, clinical outcomes, severity, and treatment in children with the most common autoimmune diseases.

## Type 1 diabetes

Chronic hyperglycemia caused by abnormalities in insulin secretion, insulin action, or both characterizes the group of metabolic illnesses known as diabetes mellitus [22]. An autoimmune disease known as type 1 diabetes promotes the loss of pancreatic beta cells, resulting in insufficient insulin production and hyperglycemia. Since type 1 diabetes is a chronic condition, it requires constant insulin replacement and intensive blood glucose level control by the patient [23]. Less than half of people with type 1 diabetes are diagnosed before the age of 15, even though type 1 diabetes accounts for more than 90% of children and adolescents with diabetes in most western countries [22].

### Impaired glucose metabolism and immune system dysregulation

High blood sugar promotes SARS-CoV-2 replication in human monocytes, and glycolysis maintains SARS-CoV-2 replication by activating hypoxia-inducible factor 1α20 and producing reactive oxygen species in the mitochondri [24]. Therefore, viral growth may be aided by hyperglycemia. The finding supports this theory that hyperglycemia or a history of Type-1 Diabetes Mellitus (T1DM) and Type-2 Diabetes Mellitus (T2DM) are independent predictors of morbidity and mortality in SARS21 patients [25]. Poor glycemic control and higher HbA1c level are associated with inflammation, hypercoagulability, and low oxygen saturation in COVID-19 patients, predicting an increased need for medications and hospitalizations and a higher mortality rate [26, 27]. Notably, those with impaired glucose regulation or diabetes mellitus frequently have glycemic deterioration as a COVID-19 complication [28].

Dysregulated immunological condition is associated with macrovascular consequences of diabetes mellitus, as hyperglycemia can impair immune function [29, 30]. Accordingly, acute respiratory virus infection(Influenza, Human respiratory syncytial virus (HRSV)) boosts IFN production and results in muscular insulin resistance in humans. This results in compensatory hyperinsulinemia, which stabilizes blood sugar levels and stimulates antiviral CD8+ T cell responses. Such compensation might not be successful in patients with impaired glucose tolerance or diabetes mellitus. It should be noted that by promoting CD8+ effector T cell activity directly, hyperinsulinemia can boost antiviral immunity. [31, 32] Reduced NK cell activity also appears in people with impaired glucose tolerance or diabetes mellitus, which may help to explain why people with diabetes mellitus are more vulnerable to COVID-19 and have a worse prognosis than people without the condition [33].

### COVID-19 outcomes in children with type 1 diabetes

There is growing evidence that children with type 1 diabetes are at a higher risk of severe COVID-19 illness (i.e., experiencing ICU admission, intermittent mandatory ventilation, or death) [12, 34]. Patients with diabetes mellitus are more likely to experience thromboembolic consequences and damage to key organs due to factors such as glucotoxicity, endothelium damage from inflammation, oxidative stress, and cytokine production [35]. In addition, antiviral medications or systemic corticosteroids, frequently used in the treatment of COVID-19 patients, may further worsen hyperglycemia [33].

It is shown that mortality rate, the relative risk of endotracheal intubation, and septic shock were increased in children with type 1 diabetes and Covid-19 than in children with Covid-19 and no Type 1 diabetes [36]. Even though well-controlled type 1 diabetes patients do not have an increased risk of infection. Special attention should be given to patients with poor glycemic control, who are at an elevated risk of infection in general due to deficient immune systems, hyperglycemia, and developing diabetic ketoacidosis (DKA). Uncontrolled diabetic children are also at a higher risk of hospitalization and infection-related complications [37–44] (Table 1). For instance, several reports have addressed COVID-19 post-inflammatory response in children with type 1 diabetes, About 2–4 weeks following the onset of COVID-19 in children; There have been cases of children acquiring the uncommon but serious condition known as a multisystem inflammatory syndrome (MIS-C). A cluster of children with hyperinflammatory shock with symptoms resembling Kawasaki illness and toxic shock syndrome was described at the height of the COVID-19 pandemic. Most MIS-C cases exhibit shock-like characteristics, including cardiac involvement, gastrointestinal symptoms, noticeably raised inflammatory markers, and positive SARS-CoV-2 laboratory test results [45–49].

**Table 1:** Clinical characteristics and outcomes in children with diabetes type 1 and COVID-19

Region	Study design	Age (years; mean, median, or range)	number (female/ male)	Duration diabetes (years; (percentage of patients))	Glycemic status, HbA1c (%)	Comorbidities (%)		Main results	Ref.	
United States	observational, multisite, cross-sectional study	0–18	412 (219/193)	< 1 (10) 1–5 (51) 6–10 (27) 11–20 (12) > 20 (0)	9.2 ± 2.3	Obesity (6) Asthma (6)		The risk for hospitalization and severe outcomes for people with T1D is age dependent.	[50]	
Egypt	Single-center retrospective observational study	9.29 ± 3.25	7	2.29 ± 1.38	11.1 ± 1.1%	0		- The prevalence of severe DKA increased due to delayed hospital admission or the effect of COVID-19.	[51]	
India	Telephonic survey	0–18	44	Not available	Not available	Not available		- Children with T1DM in the second wave of COVID-19 experience mild disease.	[52]	
India	Case report	13	1 (1/0)	Not available	8.72%	Not available		- The occurrence of rhino-orbito-cerebral mucormycosis (ROCM) in pediatric diabetic patients who have no prior history of steroid exposure suggests that hyperglycemia is the primary risk factor for the disease.	[41]	
Italy	Observational study	13.63 (8–17)	11 (7/4)	Not available	8.52%	Not available		- When infected, children and adolescents experience a milder clinical course that is typically asymptomatic and has a decreased risk of hospitalization.	[53]	
Saudi Arabia	Case report	11	1 (0/1)	Not available	Not available	Not available		- Kawasaki's presence-like symptoms should be cause for concern to think over MIS-C in children.	[46]	
USA	Surveillance study	0–18	266 (133/133)	< 1 (11) 1–5 (52) > 5 (36)	11.78%	Not available		- Elevated A1c levels are a significant risk factor for children with type 1 diabetes and COVID-19, and diabetic ketoacidosis (DKA) was the most frequent negative outcome.	[43]	
The majority were from the UK (16.3%), the USA (9.3%), and India (7%)	Observational study	0–18	25	< 1 (8) 1–5 (32) 5–10 (48) > 10 (12)	7.6 ± 1.6%		Not available	- The majority of children with COVID-19 and diabetes had only mild to moderate symptoms. However, some reported delayed diagnoses and a higher prevalence of DKA.		[27]
Israel	Observational Study	16.9 ± 5.3	132	7.8 ± 5.3	7.7%	32 (24.2)		- Young people with pre-existing T1D have a modest COVID-19 infection. - Age and higher glucose levels during COVID-19 infection were linked to a longer illness course.	[37]	
USA	Case report	16	1 (1/0)	3	11.4%	Asthma, controlled seizures		- In type-1 diabetics with COVID-19, the severity of DKA is likely multifactorial. -In children who appear with unexplained severe DKA, clinical suspicion of COVID should be increased.	[44]	

## Rheumatic diseases

One of the most prevalent chronic disorders in children is rheumatic disease. They often affect several organ systems and are complicated. Juvenile idiopathic arthritides are the most prevalent subgroup of pediatric rheumatic illnesses, followed by juvenile-onset systemic lupus erythematosus (SLE), and less common conditions such as juvenile dermatomyositis, primary vasculitides, and scleroderma [54].

Rheumatic disorders are related to a higher infection risk than the general population without the disease [55]. The primary cause is a general immune system impairment common to all autoimmune diseases, which depends on the disease activity level [56]. Studies revealed that active disease and receiving immunomodulatory drugs, i.e. corticosteroids and non-steroidal anti-inflammatory medications, are significant risk factors for an increased rate of infection [56–62]. Even when patients with rheumatic disorders stop receiving their established immunomodulatory and anti-inflammatory medicines, these treatments may negatively impact their immunological system. In the early phases of COVID-19, the diminished body's immune response and antiviral defense followed by immune-suppressant agents may harm the course of the infection. However, later on, it might also support and even help to avoid cytokine storm syndrome in the severe course of COVID-19 [63]. In addition, some other drugs administered in rheumatic diseases, like hydroxychloroquine, methotrexate, and tocilizumab, have not been associated with an increased risk of respiratory infections and even might have a therapeutic role in the treatment of COVID-19 [64, 65].

On the other hand, data have shown gene mutations in some rheumatic diseases that may lead to the cytokine storm syndrome associated with COVID-19 [66, 67]. Sawalha et al. reported that epigenetic dysregulations in SLE resulted in angiotensin-converting enzyme 2 expressions (ACE2) overexpression, and might cause a specific susceptibility to COVID-19 [17]. Comorbidities, which commonly exacerbate Rheumatoid Arthritis (RA), are another critical factor in determining the risk of infection [68]. Chronic obstructive pulmonary disease (COPD), interstitial lung disease, renal failure, diabetes mellitus, and cardiovascular disease are all concurrent conditions linked to an increased prevalence of infections in RA [62, 69].

### COVID-19 outcomes in children with rheumatic diseases

Given all factors modifying the COVID-19 susceptibility and severity in patients with rheumatic diseases, children are more unlikely to have serious rheumatic disease course; hence, they are less likely to develop severe COVID-19 infection solely because of their underlying rheumatic disease [61, 70]. In conclusion, further studies are warranted to better clarify COVID-19 severity in children with rheumatic diseases [58–61, 70].

To date, little data are available on rheumatic diseases and COVID-19, particularly among children. However, a meta-analysis showed that immune-compromised situations such as rheumatic diseases requiring immunosuppressive treatment were not a risk factor for more severe COVID-19 disease courses. Furthermore, those with and without rheumatic and musculoskeletal diseases (RMD) had nearly the same rate of hospitalization, ICU admission, and mechanical ventilation. In contrast, the mortality rate was increased in patients with RMDs (Odds ratio(OR) 1.74 [95% Confidence interval(CI) 1.08–2.80]) [71].

Several studies showed that the clinical course of COVID-19 was mild in children with rheumatic disease, with most of them being asymptomatic, regardless of whether immunosuppressive therapy was maintained or discontinued. However, there were some other studies and case report that documented severe outcomes and complications in children [11, 58–61, 72–79] (Table 2). Medium/high-dose corticosteroids, the use of mycophenolate and rituximab, and significant immunosuppression were found to be hospitalization risk factors in a case series investigating pediatric patients with rheumatic illnesses and laboratory-confirmed COVID-19. High-risk signs for admission were fever, dyspnea, chest pain, and rash. The requirement for hospitalization may be influenced by the activity and flare of rheumatic diseases [75]. In patients with underlying risk factors for hyperinflammation, COVID-19 may cause mortality regardless of the administration of biological Disease-modifying antirheumatic drugs(bDMARD) [76].

**Table 2:** Clinical characteristics and outcomes in children with rheumatic disease and COVID-19

Region	Study design	Age (years; mean or median)	Rheumatic diseases	number (women-men)	Main results	Ref.
Italy	Observational, survey based study	13 (4–20)	Chronic rheumatic diseases (juvenile idiopathic arthritis(JIA), chronic uveitis, autoinflammatory disease(AID), recurrent pericarditis, and other chronic rheumatic diseases)	123 (83–40)	- No patient needed hospitalization or to interrupt receiving therapy. - Treatment with bDMARD does not appear to raise the incidence of respiratory or life-threatening SARS-CoV-2 sequelae.	[59]
Turkey	Cross-sectional study	12.86 ± 4.76	Familial Mediterranean fever (FMF), JIA, juvenile systemic lupus erythematosus (jSLE)	149 (90–59)	- It's found that taking immunosuppressive drugs like bDMARDs or combination DMARDs won't make patients more likely to get severe SARS-CoV-2. - There was no correlation between IgA and IgG positive and age, sex, underlying rheumatic illnesses, or receiving therapies.	[60]
Spain	Observational longitudinal study	11.88 ± 4.04	Oligoarticular or polyarticular JIA, SLE, systemic JIA, monogenic autoinflammatory syndrome	77 (55–22)	- There was no correlation between the COVID-19 results and the categories of rheumatic diseases. - Risk factors for hospital admission include comorbid conditions and glucocorticoid therapy. - Thirty patients (38.96%) had no symptoms, forty-one (53.25%) had mild-moderate COVID-19, six (7.79%) needed hospital admission, one needed critical care, and no one died.	[79]
Spain	Cross‑sectional study	11.8 ± 4.5	JIA, SLE, Other	105 (76–29)	- The COVID-19 clinical course was mild, with more than one-third of individuals being asymptomatic. - Oral corticosteroids and increased underlying disease activity are risk factors for SARS-CoV-2 infection.	[72]
Germany	Observational, Survey based study	14	JIA, AID, Connective tissue disease(CTD)	76 (40–36)	- Seventy six percent of the population showed signs of COVID-19. - Regardless of whether the immunosuppressive medication was continued or stopped, 44 of 46 symptomatic patients (96%) had a mild disease course. - One of the two patients who were admitted to the hospital passed away. - The underlying rheumatic and musculoskeletal disease activity did not worsen or did so just slightly.	[80]
Turkey	Retrospective Study	13.06	AID, JIA, CTD, Vasculitis	658 (339–319)	- The risk of hospitalization or symptomatic infection was not found to be higher in patients receiving biological therapy.	[61]
Ukraine	Observational study	12.3	JIA	51(26–25)	- Patients with the systemic arthritic disease had higher COVID-19 occurrence rates. (OR = 6.1667, 95% CI: 1.2053–31.5511, p = 0.0289). - Severe COVID-19 course was not seen in JIA patients, particularly those on immunosuppressive therapy.	[73]
Turkey	Observational, survey based study	12 ± 4.7	JIA, AID, CTD, vasculitis	414	- None of them—not even the confirmed case—exhibited any serious symptoms. - Since more than half of the patients with household exposure were asymptomatic, they did not need to be hospitalized. - It appears that the COVID-19 infection did not result in any significant symptoms or problems.	[74]
Turkey	Single-center, prospective, observational study	15.4 ± 1.5	JIA, periodic fever syndrome, Colchicine-resistant FMF, other	41 (15–26)	- Paediatric rheumatic disease patients can experience an effective humoral response after two dose regimens of the BNT162b2 mRNA vaccination without having to stop their regular therapies.	[81]
USA	Single-center case series	16 (14–18)	JIA, SLE, Juvenile Dermatomyositis	55 (43–12)	- Hospitalization risk factors were the use of mycophenolate, rituximab, and medium/high dose corticosteroids, as well as severe immunosuppression. - Dyspnea, chest discomfort, rash, and fever were considered to be high-risk indicators for hospitalization. - The occurrence of a rheumatic illness flare-up may necessitate hospitalization.	[75]
Turkey	Retrospective Study	12.3	JIA, systemic auto inflammatory diseases, vasculitis, chronic recurrent multifocal osteomyelitis, Sjögren’s syndrome	39 (22–17)	- Regardless of the administration of bDMARDs, COVID-19 may become severe or result in mortality in patients with background risk factors for hyperinflammation.	[76]
Poland	Case–Control Study	12(2–18)	JIA	62	- JIA patients do not exhibit a higher seroprevalence of anti-SARS-CoV-2 antibodies than healthy individuals.	[82]
Turkey	Single-center, retrospective study	12	JIA, FMF, systemic autoinflammatory diseases	87(37–50)	- Children with rheumatic diseases do not have a higher chance of developing severe COVID-19. - The disease's severity is unaffected by biological treatment.	[83]
USA	Cross-sectional study	16 (2–25)	JIA, CTD, Vasculitis, Juvenile dermatomyositis, Uveitis	262(186–76)	-The most prevalent symptoms were fever, exhaustion, and cough. - No patients required hospitalization due to severe COVID-19.	[78]
Turkey	Retrospective and multicenter study	12.87 ± 4.69	JIA, AID, FMF, other	113(72–41)	- The overall hospitalization rate was 21.2%, and ambulatory care was provided to 78.8% of the patients. - Compared to ambulatory patients, hospitalized patients were younger, had shorter disease durations, and used steroids more frequently. - Under bDMARDs, a worsening of the course of either COVID-19 or the current illness was not observed.	[58]

Moreover, numerous rheumatic disorders were identified in survey-based cohort research in children, and immunosuppressive therapy does not appear to increase the risk. On the other hand, abruptly stopping these medications could result in clinical instability and aggravation of the underlying condition. Additionally, one should be cautious to avoid COVID-19-related disease exacerbations [74].

In summary, there is controversial evidence, but it appears that comorbidities, higher disease activity, and oral corticosteroids can be risk factors for severe SARS-CoV-2 infection.

## Autoimmune neurological diseases

Although COVID-19 is typically thought of as a respiratory disease, numerous reports have noted that COVID-19 also has neurologic symptoms [84]. An alarming number of neurological symptoms associated with COVID-19 have been reported, ranging from minor ones like headaches and myalgia to serious ones like stroke and encephalopathy [85]. With 45.5% of those with severe COVID-19 infection experiencing neurologic symptoms compared to 30.2% of those with non-severe infection, severe infection was linked to a greater prevalence of neurologic manifestations [84]. Compared to the adult population, fewer neurologic consequences of COVID-19 have been identified in pediatric patients. In children, most symptoms are restricted to headache and/or loss of smell or taste [86]. There are, however, case reports documenting more serious neurologic issues, such as encephalitis, seizures, and cerebrovascular stroke in children [86–95].

ACE2 is expressed on the blood–brain barrier (BBB)’s vasculature and facilitates SARS transport by targeting the spike protein [96]. The olfactory bulb and the cribriform plate are direct entry points for SARS-CoV-2 into the central nervous system, where it can cause neuronal injury and prevent oligodendrocyte differentiation and remyelination. The loss of microglial cells impairs the removal of myelin debris, and the release of neurotoxic soluble substances from activated astrocytes contributes to pathological reactions. In addition, SARS-CoV2 can trigger the development of CNS-specific lymphocytes through molecular mimicry. It is crucial to note that the BBB is damaged from the inside and outside, making it easier for peripheral immune cells to infiltrate the CNS parenchyma. This is because of the neurotropic and hematogenous routes of infection and disease impingement on the BBB [97]. The potential for molecular mimicry and the stimulation of auto-reactive T cells against myelin is also worthy to note. Through this possible mechanism, SARSE-COV 2 can cause disease exacerbation or develop new other autoimmune diseases [98].

It is possible that COVID-19's invasion of CNS could make any existing neurologic damage worse [99]. This is particularly relevant in the setting of neurological disorders with an autoimmune base, like multiple sclerosis (MS), as patients with these conditions already have increased cytokine levels and are regularly taking disease-modifying treatments (DMTs) that can impair their immune systems [100]. When the BBB breaks down, immune cells infiltrate and help to promote demyelination. COVID-19 causes vascular injury and elicits a generalized inflammatory response, which may exacerbate the breakdown of the BBB and accelerate the course of multiple sclerosis (MS) [99]. The most prevalent acute CNS demyelinating illness, acute disseminated encephalomyelitis, primarily affects younger adults and children.

Acute disseminated encephalomyelitis (ADEM) is an inflammatory demyelinating disease of the central nervous system that primarily affects children under 10 years old and is more common in males. The disease typically develops 1–2 weeks after infections or, less frequently, after vaccinations. It affects multifocal areas of the white matter, rarely the gray matter, and the spinal cord [101]. In patients with a genetic predisposition, ADEM pathophysiology is hypothesized to be connected to the antigenic mimicry theorem [102]. To put it another way, a recent viral or bacterial infection stimulates the immune system, particularly the T-helper 2 (Th2) cells and neutrophils, which causes excessive production of cytokines and chemokines [103, 104]. Infection with COVID-19 has been associated with cytokine storms that harm the central nervous system [105]. Thus, COVID-19 infection could exacerbate an already ongoing ADEM illness. Nevertheless, this does not negate the possibility that COVID-19 systemic infection can induce ADEM on its own [106].

The adverse events following the immunotherapies are the primary concern with COVID-19 in all neuro-immunological diseases. One important component is the systemic, long-term alteration of the immune response brought on by corticosteroids, immunosuppressants, or DMT medications [107]. DMTs can be grouped into the following categories, albeit classification is not entirely correct, as different DMTs have diverse modes of action: 1) Interferon β-1 (IFN-β1), glatiramer acetate (GA), and fumarates (e.g., dimethyl fumarate) are immunomodulators. 2) Cell trafficking changes are caused by agents like S1P receptor modulators (e.g., fingolimod) and natalizumab, an anti-4-integrin antibody). 3) cell depletion is caused by anti-CD20 antibodies (e.g. teriflunomide) [108, 109].

Different classes of drugs are associated with different levels of risk. Compared to non-MS individuals of the same age and sex, MS patients who received GA and IFN-β1 via DMTs had a 50% higher risk of all serious infections (defined as an infection requiring hospitalization). the anti-CD20 antibody rituximab In comparison to GA and IFN-β1 dramatically increased the rate of overall severe infection in MS patients [110]. However, some believed that DMTs might protect against COVID-19 by reducing the cytokine-storm-like reactions [110, 111]. Additionally, several DMTs (such as GA, fumaric acid, and fingolimod) are linked to an upsurge in the expression of circulating natural killer cells, which could lead to a more effective defense against COVID-19 [112].

**Fig. 1:**
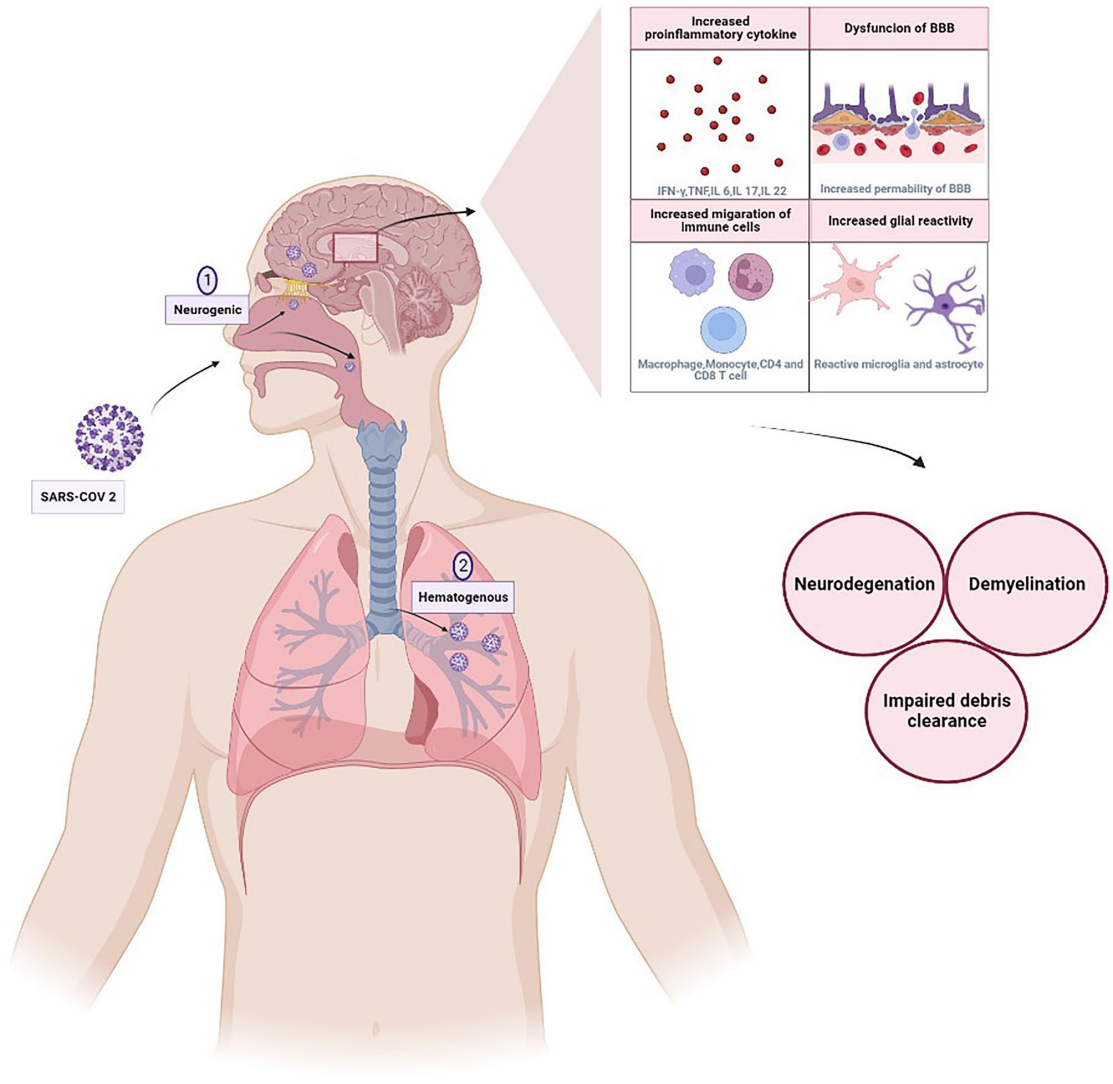
SARS-COV 2 potential for neurological disease exacerbation. Two potential neural pathways for SARS-COV 2 entry into the brain are by infection of the olfactory bulb or interactions with the eyes and oral mucosa. SARS-CoV-2 may transmit infection along blood–brain barrier (BBB) endothelial cells, blood-cerebrospinal fluid barrier epithelial cells in the choroid plexus, or it may enter the body through the CNS by making use of inflammatory cells as a "Trojan horse" [113]. Expression of IL-6, TNF-α, and other proinflammatory cytokines in a cytokine storm increased due to Viral presence, also glial inflammatory response may lead to damaged oligodendrocytes and BBB disruption, providing a second way for CNS invasion and lymphocyte infiltration [114]

It is impossible to draw definite conclusions regarding the effect of medication and the severity of the infection in these patients. Figure 1 depicts the possible neurological damage as a result of COVID-19 infection (Fig. 1).

### COIVD-19 outcomes in children with autoimmune neurological diseases

Patients with autoimmune neurological illnesses diagnosed with COVID-19 are currently the subject of extensive data gathering. However, these samples have not yet reached statistical significance [115]. Previously, viral infections were linked to several autoimmune neurological conditions, most notably MS [116]. Relapses and/or worsening neurological symptoms are common effects of infections, which also significantly increase morbidity and contribute to the exacerbation of the disease [117, 118]. Upper respiratory viral infections, including infections with coronaviruses, have been shown to raise the possibility of relapse in people with MS [119, 120]. Coronaviruses account for 10 to 30 percent of these infections [119, 121].

Among the 404 patients with MS and SARS-CoV-2 infection enrolled in the prospective cohort study conducted by Garjani et al., 2021, 230 patients (57%) reported worsening symptoms of their MS. 207 patients reported a worsening of pre-existing symptoms, 82 reported the emergence of new MS symptoms, and 59 reported both [122]. To date, outcomes of COVID-19 in pediatrics with Autoimmune neurological diseases are largely unknown. Children frequently experience mild illness, because age is an established risk factor for serious illness [123].

In a small subgroup of patients with pediatric-onset MS who had been exposed to the SARS-CoV-2 virus or who had developed COVID-19, neither group required hospitalization nor respiratory support and reported no or mild symptoms. Additionally, they stated that comorbidities, neurological impairments, and DMTs had no influence on the outcomes of COVID-19 [115].

It has also been shown that COVID-19's extreme immunological activation and systemic stress may contribute to a higher frequency of relapses in people with MS [124, 125]. Also, The number of relapses in the pre-defined at-risk period was compared with the previous two years in a retrospective analysis of 41 patients with relapsing–remitting MS. The results revealed that COVID-19 may cause an exacerbation of MS [119].

Interestingly, children account for 30% of COVID-19-related ADEM cases reported so far. MIS-C, an entity associated with ADEM-like illnesses, is supposed to manifest 10 to 54 days after COVID-19 infection [93, 126, 127].

Henriques et al. reported that a 12-year-old girl with ADEM, severe cervical spinal cord myelopathy, and concurrent COVID-19 infection, finally evolved with partial clinical and neurological improvement and was subsequently discharged [128]. There were cases of younger children as well, for instance, a 17-month-old child who developed ADEM two weeks following COVID-19 pneumonitis and was finally able to fully recover after undergoing an intense immunomodulatory medication course [129]. The prognosis can vary. For instance, out of 38 pediatric patients with neuro-COVID-19, four passed away very quickly from fulminant systemic co-infections, and one child had a presumptive diagnosis of ADEM [126]. The definite underlying determinant of the prognosis is not yet discovered. However, investigations are ongoing. For example, children with monophasic or relapsing CNS demyelinating illnesses that include the optic, cerebral, and spinal structures and have overlapping clinical symptoms are reported to have myelin oligodendrocyte glycoprotein (MOG) antibody-associated disorders [130, 131]. In rare cases, COVID-19 infection might potentially cause an anti-MOG illness relapse [132].

Studies showed that the majority of COVID-19-positive neuromyelitis optica spectrum disorder (NMOSD) patients had moderate illness manifestations. However, compared to the general population, NMOSD patients had significantly greater probabilities of being admitted to the hospital and the critical care unit. Additionally, SARSCoV2 infection has increased the incidence of NMOSD relapses. Comorbidities such as Hypertension, obesity, diabetes, and dyslipidemias are the only predictor found to be associated with a worse COVID-19 outcome in patients with NMOSD (OR = 6.0, 95% CI: 1.79–19.98) [133]. However, some other reports show patients present similar incidence, risk factors, and outcomes for COVID-19 as the general population [134–136].

Treatment risks and advantages for patients with these disorders must be weighed against the degree to which disease-modifying medications restrict antiviral host immunity. As was previously noted, several immunotherapies are available for different neuroimmunological diseases [116].

Although there are controversial data, it can be concluded that higher disability status pre-existing comorbidities, patients with progressive forms, and a longer disease duration increased the risk of a more severe COVID-19 course of disease [122, 136, 137].

## Other autoimmune disorders

### COVID-19 in children with inflammatory bowel diseases (IBD)

Among individuals with inflammatory bowel diseases (IBD), COVID-19 susceptibility and disease progression are yet unknown, and there is a scarcity of epidemiological data on the subject [138]. We should anticipate a higher chance of SARS-CoV-2 infection and/or a worsening prognosis in patients with IBD. These patients have attenuated immune systems and an increased risk of infection due to the immunosuppressive medications they take [139]. Also, excessive cytokine production raises the ACE2 [140]. Thus, the gut mucosa of these patients exhibits an increased expression of ACE2 [141] and a subsequent rise in serum ACE2 levels (as well as Ang1–7 and the ACE2: ACE ratio) [142]. In the blood, this might serve as a protective factor by serving as the virus receptor’s rival which lowers the viral load that would otherwise infect the host. As both mucosal and serum ACE2 expression seems to be elevated in IBD and it is an important question as to how exactly ACE2 affects COVID-19 unfavorable consequences in IBD patients given its crucial function in enabling the virus' entrance into host cells [141–143]. In addition, given that immunosuppressive medication is frequently used by IBD patients, and subsequent immune system dysregulation, distinct COVID-19 forms in these individuals compared to the general population could be anticipated [144].

Few papers that have been published so far do not indicate an elevated incidence of COVID-19 in people with pediatric inflammatory bowel illness [139]. According to the Surveillance Epidemiology of Coronavirus under Research Exclusion (SECURE)-IBD registry, there have been 1760 instances of COVID-19 in IBD patients (85 of them are registered in Italy), of which 497 required hospitalization (28%) and 63 passed away (4%) [145]. According to a systematic study of IBD patients with COVID-19, 28 (11.4%) out of 246 patients required ICU care, 26 (3.7%) out of 697 patients required mechanical breathing, and 29 (3.8%) out of 796 patients died because of COVID-19 [146]. A SECURE-IBD registry preliminary report on the initial 525 patients from 33 countries reported that advanced age, the presence of at least two comorbidities, the administration of systemic steroids or sulfasalazine/5-aminosalicylate as risk factors of a worse course of the SARS-Cov-2 virus infection [147]. According to another study by Carparelli et al., out of 600 IBD adult patients analyzed, including pediatric patients, COVID-19 was diagnosed in 25 patients, none of whom were under the age of 18 years. This suggests that the incidence rate of COVID-19 is lower in children. Symptoms were frequently missing or barely noticeable. There was no recorded death [148].

A greater prevalence of hospitalization was associated with the use of corticosteroids (29 vs. 8%) and sulfasalazine/mesalazine medication (57% of inpatients vs. 21% of outpatients). In addition, exactly like in adults, the use of TNF antagonists alone was associated with a lower chance of hospitalization in pediatric patients (7% of hospitalized patients vs. 51% of non-hospitalized). [149]. Severe COVID-19 may be associated with clinically active IBD, especially in younger people. Controlling IBD disease is essential to prevent harmful COVID-19 effects, particularly through medication compliance and methods to lower the chance of COVID-19 infection in individuals with active IBD (i.e. isolation, vaccination) [150]. Moderate/severe disease activity was linked to an increased risk of hospitalization in a study of 209 pediatric IBD patients from the SECURE-IBD registry. However, the sample size was too limited to allow for the adjustment of confounding factors, and the outcomes like ICU admission and death were too few to examine. Additionally, it has been noted that children receiving biologics and/or other immune-suppressive treatments had a low risk of developing severe COVID-19 [149].

MIS-C, also called pediatric multi-system inflammatory syndrome temporally related to SARS CoV-2 (PMIS or PIMS-TS), is a potentially serious illness in children that appears to be a delayed, post-infectious complication of COVID-19 infection. The most common symptoms in children with PIMS-TS/MIS-C are gastrointestinal ones. Hence it could be difficult to distinguish this condition from an inflammatory bowel disease (IBD) flare-up. Data have demonstrated that in patients with underlying IBD, the primary cause of treatment and diagnostic difficulties in PIMS-TS/MIS-C is the similarity of clinical presentations. Children who have recovered from PIMS-TS/MIS-C require long-term follow-up to determine their risk of developing autoimmune diseases [151]. Other studies showed that despite continued immunomodulatory therapy, children with IBD and symptomatic or asymptomatic SARS-COV-2 infection could establish a protective humoral response against SARS-CoV-2 that is comparable to their peers without the disease [152, 153]. IBD treatment should not be stopped during the pandemic, according to current recommendations, as the risk of exacerbations outweighs the risk of any COVID-19 consequences [154, 155]. This is particularly for children, as in the first wave of the COVID-19 pandemic, illness flare-ups occurred in 21–23% of pediatric patients who had discontinued or temporarily paused their biological treatment [156].

Although there is a growing interest in the connection between COVID-19 and IBD, there are still some problems that need to be addressed in further in-depth research with larger patient cohorts, particularly in pediatric populations. The risk of infection or the severity of the disease does not seem to be higher for IBD patients. However, further research is needed to attain more certainty because the findings of the published studies so far are in disagreement.

### COVID-19 in children with celiac disease

Given the strong expression of ACE2 and TMPRSS2 in the intestinal enterocytes, an increasing body of evidence supports the intestinal tropism of SARS-CoV-2. In fact, COVID-19 stimulates the production of a "cytokine storm" in the intestinal mucosa, which results in epithelial destruction and enhanced barrier permeability, allowing gliadin to pass through the intestinal lamina [157]. Impairment of the intestinal barrier causes the translocation of microbial elements, including microbial-associated molecular patterns (MAMPs), which in turn trigger an inflammatory immune response by TLR-expressing cells of the mesentery fat (primarily macrophages and adipocytes) and can thus enter the bloodstream [158]. These results support the idea that intestinal cells may contribute to an increase in SARS-CoV-2 viremia [157].

The etiology of many autoimmune illnesses has been linked to increased intestinal permeability because this particular permeability results in irregularities in the movement of components that might elicit certain autoimmune reactions [159]. Gliadin causes an autoimmune systemic illness called CD in people who are genetically prone to it (HLA DQ2 and DQ8) [160], which is associated with a higher risk of viral infections [161]. One of the key roles in the pathogenesis of CD is the disruption of the intestinal barrier [160]. CD Children and adults have both been described as being infected during the COVID-19 pandemic [162, 163]. There have been new proposed CD diagnostic methods [164], worse clinical status [165], and a decreased diagnostic rate described in CD patients during the COVID-19 pandemic [166].

CD patients, particularly untreated individuals, may be more susceptible to infections as well as viral diseases. It has been postulated that increased expression of CD4, CD25, and FOXP3 as anti-inflammatory markers in CD patients might be of benefit to reducing the severity of COVID-19 disease. Nevertheless, because of their elevated expression of IL-6, untreated CD patients may be more susceptible to severe COVID-19 if they contract the SARS-CoV-2 virus [167]. Future research involving CD patients infected with COVID-19 will need to demonstrate that increased expression of anti-inflammatory markers in these patients can reduce the severity of COVID-19. It is shown that CD patients with COVID-19 do not have a higher risk of death or hospitalization [168]. In Sweden, a recent population-based study of CD patients revealed no elevated risk of COVID-19-related outcomes [169]. None of the 387 pediatric CD patients in an Italian study experienced respiratory failure, developed pneumonia, needed oxygen therapy, or needed to be hospitalized. Additionally, compared to the general population, a significant rise in COVID-19 incidence was not indicated [163]. Hospitalization and mortality rates for CD patients who also have COVID-19 were 12% and 2.5%, respectively, according to a global registry of health professionals [168]. Moreover, increased age and new gastrointestinal symptoms in CD patients may raise their risk of adverse COVID-19 outcomes, similarly the patients without CD [170, 171].

### COVID-19 in children with psoriasis

Psoriasis is a papulosquamous, immune-mediated, chronic skin condition that affects 125 million individuals worldwide [172]. A well-known cause of psoriasis, particularly guttate psoriasis in children, is viral and bacterial infections which can either cause psoriasis de novo or ignite an aggravation of psoriasis [173–175]. Similarly, SARSCoV-2 may exacerbate psoriasis [176–178]. The skew towards an excessive inflammatory cytokine milieu in psoriasis, notably tumor necrosis factor- and IL-17, may be responsible for the severe inflammation and tissue destruction seen in bacterial and viral infections  [179, 180]. It was found that pro-inflammatory cytokines produced in excess cause immune response dysregulation and pathological inflammatory alterations connected to septic shock [181].

Following 69,315 patients with psoriasis, Yiu et al. [182] found a 36% increased risk of being hospitalized and a 33% increased risk of death because of serious infections among patients with psoriasis relative to matched controls. A controlled retrospective cohort study followed 25,742 psoriasis patients and revealed a higher chance of acquiring severe infections (adjusted hazard ratio 2.08; 95% CI 1.96–2.22) [183]. Similarly, a cohort of 199,700 psoriasis patients was found to have an increased risk of serious infections (adjusted hazard ratio 1.21; 95% CI 1.18–2.23) [184].

Apremilast is an oral phosphodiesterase-4 inhibitor approved for the treatment of chronic plaque psoriasis, psoriatic arthritis and Behcet’s disease [185]. Given that apremilast inhibits the expression of pro-inflammatory cytokines known to be released with SARS-CoV-2, such as tumor necrosis factor-alpha (TNF a), interleukin (IL)-17, and IL-23 [186], it is possible that using this oral small molecule may reduce the risk of a cytokine storm associated with SARS-CoV-2 infections. Patients using apremilast may therefore have a lower risk of experiencing serious complications with COVID-19  [187, 188]. Apremilast exhibited the lowest SARS-CoV-2 infection rate when compared to biologic treatments in a Spanish cohort of psoriasis patients [189]. Adults are the focus of the majority of published data on the usage of systemic psoriasis therapies and COVID-19 outcomes. Old age, male sex, non-white ethnicity, and comorbidities (mostly chronic lung diseases) are the same risk factors for worse COVID-19 outcomes in adult psoriasis patients as they are in the general population, according to Mahil et al. (PsoProtect registry) [190].

Biologic treatments, cyclosporine, and methotrexate, among other systemic psoriasis medications, are known to make both adults and children more susceptible to infections [191]. It has been revealed that compared to children receiving biological medications, children receiving non-biologic systemic therapy (P = 0.02) and those not receiving systemic treatment (P = 0.006) had significantly longer COVID-19 symptom durations (6.5 days on average). Additionally, it is noted that the six hospitalized children were treated with non-biologic systemic medications, methotrexate in particular (P = 0.03), more frequently than the others (P = 0.01). In 17 patients (15.2%) after COVID-19, psoriasis got worse. In the month after COVID-19, nine children (8%) developed psoriasis (P = 0.01) [192]. The COVID-19 treatment course in psoriasis patients of all ages has been evaluated by the PsoProtect registry. It demonstrated that utilizing biologics did not make psoriasis patients more likely to be hospitalized. Few pediatric patients were included in PsoProtect and other investigations, and no pediatric-specific subgroup analysis has yet been carried out [39, 178, 181, 187–189].

In a French study, the first and second waves of the pandemic were separately analyzed as they examined the COVID-19 outcome in individuals with psoriasis from the national health insurance database. For both waves, no difference in mortality between patients receiving biologic vs. non-biologic systemic medications was reported. During the initial wave, they observed an elevated risk of hospitalization for individuals using non-biologic systemic medications [193].

Studies observed no evidence of an elevated risk of severe COVID-19 in patients receiving biologic therapy in this multinational registry of psoriatic infants, children, and adolescents from 14 countries across three continents who developed COVID-19. Biologic treatment for psoriasis in children does not seem to increase their chance of getting COVID-19 severe. For some, COVID-19 caused psoriasis to develop or worsen in people who already had it [192].

## COVID-19 treatment in children with autoimmune disorders

Due to the increased risk of COVID-19 infection, the likelihood of serious COVID-19 consequences, and the potential for pre-existing illness flares, patients with autoimmune diseases and their caregivers are concerned about how well these patients are being treated. The underlying autoimmune diseases’ pathophysiology and severity, as well as the received medications, can impact the Covid-19 outcomes in patients with autoimmune diseases [194].

Children with autoimmune disorders may present different symptoms and require specific prevention plans or therapies, and medical professionals must remain informed about these treatments to provide the best care to their patients. For instance, while hydroxychloroquine has been widely administered for COVID-19 treatment, the management of SARS-CoV-2-infected T1DM patients has demonstrated that it can lead to a reduction in insulin breakdown and, subsequently, hypoglycemia [195]. Nevertheless, antiviral medications like ritonavir and lopinavir have been shown to decrease glycemic control and result in hyperglycemia [196]. Significant hyperglycemia can also result from the use of glucocorticoids, which are widely used as a part of the COVID-19 treatment regimen for hospitalized patients [195].

Outpatient SARS-CoV-2 treatment includes Monoclonal antibodies, remdesivir, and oral medicines like nirmatrelvir/ritonavir and molnupiravir [197–200]. Studies have shown that patients with systemic autoimmune rheumatic diseases (SARD) can benefit from receiving monoclonal antibodies and also from oral antiviral medications. However, there are some concerns raised about a higher risk of COVID-19 rebound following the administration of the latter [201].

Despite these concerns, a cohort study of SARD patients with COVID-19 found that a rebound has occurred in only 8% of SARD patients who received oral outpatient therapy. Furthermore, none of these patients ended up demonstrating severe symptoms or were hospitalized as a result of the COVID-19 rebound. Compared to no outpatient treatment, antiviral or monoclonal antibody outpatient treatment was linked with 88% lower odds of severe COVID-19 infection [201]. Another investigation revealed that both oral antiviral therapies (molnupiravir and nirmatrelvir/ritonavir) had positive outcomes and adequate safety profiles among a high-risk SARD population [202].

On the other hand, Many immunomodulatory therapies that are routinely used for autoimmune diseases, including tocilizumab, which is often used to treat autoimmune diseases, may help control the immune response against SARS-CoV-2 [203]. Antimalarial medications chloroquine and hydroxychloroquine, have immunomodulatory properties and are also used to treat autoimmune conditions such as SLE and RA. Both medications are reported to prevent the fusion of the SARS-CoV-2 coronavirus with host cell membranes. Moreover, chloroquine prevents the cellular ACE2 receptor from being glycosylated, which might prevent SARS-CoV-2 from attaching to its cellular receptor. Both chloroquine and hydroxychloroquine can also inhibit the movement of SARS-CoV-2 from early endosomes to endolysosomes in vitro, which is necessary for the release of the viral genome [204, 205]. One study on immunosuppressive medication in psoriasis patients with COVID-19 found that those taking apremilast have a reduced incidence of infection. It is important to investigate the bidirectional impact of COVID-19, as an immunomodulatory acute condition, and immunomodulatory drugs. Studying these can help to investigate if continuing these drugs for autoimmune patients, infected or at risk of COVID-19, would be safe. These investigations, for instance in the case of chloroquine and hydroxychloroquine, might even lead to the usage of certain immunomodulatory drugs for COVID-19 treatment in the normal population [189].

In conclusion, although it is well known that those with pre-existing autoimmune disorders are at higher risk for severe complications from COVID-19, it is important to conduct further studies to understand the potential differences of common anti-COVID-19 therapies in the treatment of the virus in this underrepresented pediatric population.

## Future perspective of pediatrics autoimmune diseases post-COVID-19 pandemic

COVID-19 and comorbidities created a vicious cycle that dramatically increases mortality and morbidity in affected patients. The pathogenesis of both COVID-19 and autoimmune diseases entails an active immune system response. In addition to lymphopenia, COVID-19 can affect T-cell function, upregulate inhibitory immune checkpoint expression, and exacerbate inflammatory cytokines [190]. Patients with COVID-19 may also exhibit autoantibodies, which are common in autoimmune diseases. Furthermore, following the COVID-19 infection, some patients have developed autoimmune illnesses such as Guillain–Barre syndrome or SLE [127, 206]. Actually, severe COVID-19 has been documented to result in cytokine storms due to the overproduction of pro-inflammatory cytokines such as IL-6.

**Fig. 2:**
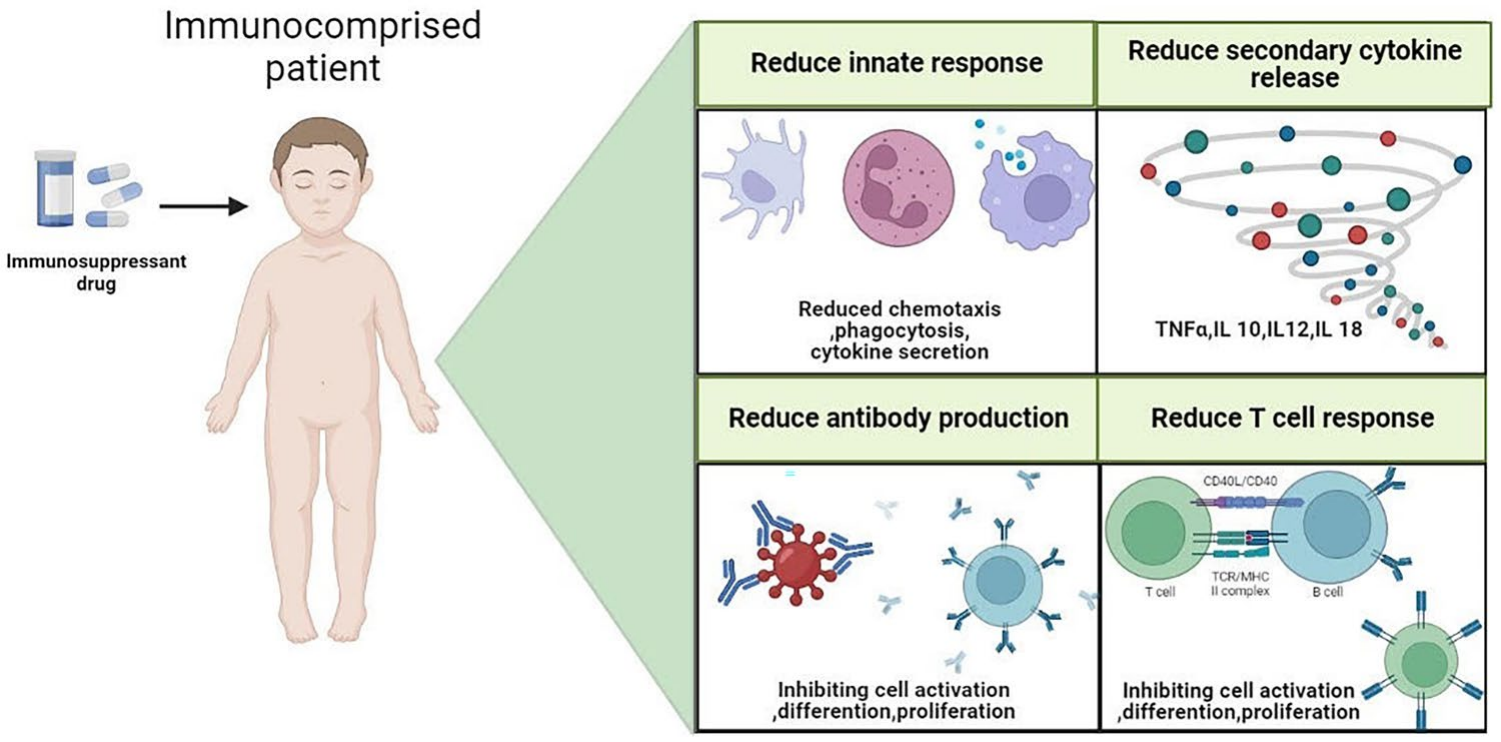
Adverse effects of immunosuppression that may influence the course of COVID-19. Reducing the immune systems' capacity to fight infections is one of the most important side effects to be concerned about in immunocompromised patients. Immunosuppressive medications have anti-inflammatory and immunomodulatory effects by preventing the synthesis of pro-inflammatory cytokines, lowering leucocyte trafficking, and causing T-lymphocyte apoptosis. These drugs stop the immune system from battling the pathogen. Hence, in the early phases of COVID-19, they could be hazardous. There is currently disagreement over the overall impact of immunosuppressive medications in patients with COVID-19

The best way to treat individuals with immune-mediated disorders that require systemic medications during the COVID-19 pandemic is a major source of concern for the medical community, for instance, regarding the fact that immunosuppressive therapy can compromise antiviral immunity (Fig. 2). In some studies, the risk of poor COVID-19 outcomes related to the use of biological medications in inflammatory illnesses such as psoriasis, rheumatologic, and intestinal diseases in people has been evaluated. Patients with severe asthma who were receiving biologic therapy had a more severe COVID-19 course than people in the general population [207]. The analysis also showed that Immune-mediated inflammatory disorders were associated with a greater rate of COVID-19 mortality and hospital admissions [208]. However, data specifically about autoimmune disorders are still needed.

Due to uncertainties in epidemiologic data on children, there is still much to learn about COVID-19 manifestations in children with autoimmune diseases. The patient might ultimately experience severe outcomes as a result of the consequences, including multiple organ failure, shock, acute respiratory distress syndrome, heart failure, arrhythmias, renal failure, and, in the worst cases, death. Consequently, better management with special consideration must be given to these patients. There is still a need for more research in order to identify the specific COVID-19 characteristics that differ between children and adults.

The preliminary results from the ongoing trials for the COVID-19 vaccination in children have demonstrated reassuring efficacy and tolerance. The balance of risk and benefit of immunization in this age group is controversial, mostly due to the relatively low risk of COVID-19 infection in children and the lack of confidence regarding the relative effects of vaccination and disease [209, 210]. The duration of the immune responses and protection provided by vaccine regimens in pediatric patients with underlying disorders call for further research, as well.

## Conclusion

The COVID-19 global pandemic posed severe health risks, particularly for people with pre-existing medical disorders. Initial concerns that patients with neuro-immune diseases were at a higher risk of developing a severe COVID-19 infection stemmed from differences in immune function caused by the conditions or treatments. Numerous studies on adults and young adolescents revealed that these groups of patients had worse COVID-19 outcomes. A relatively small number of studies reported such an observation in the pediatric age group. Given the rarity of pediatric cases, it is understandable that only case reports and studies with small sample sizes have been published.

It would be interesting to see if the presence of pre-existing immune-mediated diseases or prior use of immunomodulatory drugs influences the phenotype of COVID-19. While some studies found that patients with certain immune-mediated and autoimmune diseases had a higher risk of infection and a more aggressive course of SARS-CoV-2 infection, other studies disagreed. COVID-19 outcomes may be worse for patients receiving immunomodulatory therapies, particularly those with severe comorbidities. On the other hand, it has been proposed that the lung damage caused by SARS-CoV-2 is the result of an overactive immune system and that immunosuppressive medication may benefit some patients.

Finally, it is unclear how immune-related illnesses will affect the progression of COVID-19. The infection risk and prognosis of COVID-19 in patients with autoimmune diseases are still being debated, but patient adherence to medication regimens to prevent autoimmune disease flare-ups is strongly advised.

## Data Availability

Data sharing is not applicable to this article as no new data were created or analyzed in this study.

## Abbreviations

OR: Odds ratio
CI: Confidence interval
RMD: Rheumatic and musculoskeletal diseases
OL: Oligodendrocyte
MIS-C: Multisystem inflammatory syndrome in children
PIMS-TS: Pediatrics multisystem inflammatory syndrome temporally associated with COVID-19
HRSV: Human respiratory syncytial virus
bDMARD: Biological Disease-modifying antirheumatic drugs
MS: Multiple sclerosis
ADEM: Acute disseminated encephalomyelitis
ARP: Pre-defined at-risk period
SLE: Systemic lupus erythematosus
RA: Rheumatoid arthritis
SARD: Systemic autoimmune rheumatic diseases
TIDM: Type-1 Diabetes Mellitus
T2DM: Type-2 Diabetes Mellitus
JIA: Juvenile idiopathic arthritis
FMF: Familial Mediterranean fever
jSLE: Juvenile systemic lupus erythematosus
CTD: Connective tissue disease
AID: Autoinflammatory disease
ROCM: Rhino-orbito-cerebral mucormycosis
